# Supplementary material for: Thinking out loud, an open-access EEG-based BCI dataset for inner speech recognition
Source: Sci Data. 2022 Feb 14;9:52. doi: 10.1038/s41597-022-01147-2 (PMC8844234; doi:10.1038/s41597-022-01147-2)
Supplement: Supplementary file 1 — SUPPLEMENTARY INFORMATION [file 41597_2022_1147_MOESM1_ESM.pdf]

Supplementary Table 1. Distribution of trials in session 1. * Check the "Ad-Hoc Correction" Section for further details of the uneven number of trials in Participant sub-03.

	Run 1 - Pronounced Speech							
Participant	Up	Down	Right	Left				
sub-01	10	10	10	10				
sub-02	10	10	10	10				
sub-03	10	10	10	10				
sub-04	10	10	10	10				
sub-05	10	10	10	10				
sub-06	10	10	10	10				
sub-07	10	10	10	10				
sub-08	10	10	10	10				
sub-09	10	10	10	10				
sub-10	10	10	10	10				
	Run 2 - Inner Speech				Run 3 - Inner Speech			
Participant	Up	Down	Right	Left	Up	Down	Right	Left
sub-01	10	10	10	10	10	10	10	10
sub-02	10	10	10	10	10	10	10	10
sub-03	10	10	10	10	0*	0*	0*	0*
sub-04	10	10	10	10	10	10	10	10
sub-05	10	10	10	10	10	10	10	10
sub-06	10	10	10	10	10	10	10	10
sub-07	10	10	10	10	10	10	10	10
sub-08	10	10	10	10	10	10	10	10
sub-09	10	10	10	10	10	10	10	10
sub-10	10	10	10	10	10	10	10	10
	Run 4 - Visualized Condition				Run 5 - Visualized Condition			
Participant	Up	Down	Right	Left	Up	Down	Right	Left
sub-01	10	10	10	10	10	10	10	10
sub-02	10	10	10	10	10	10	10	10
sub-03	20*	20*	20*	20*	10	10	10	10
sub-04	10	10	10	10	10	10	10	10
sub-05	10	10	10	10	10	10	10	10
sub-06	10	10	10	10	10	10	10	10
sub-07	10	10	10	10	10	10	10	10
sub-08	10	10	10	10	10	10	10	10
sub-09	10	10	10	10	10	10	10	10
sub-10	10	10	10	10	10	10	10	10

Supplementary Table 2. Distribution of trials in session 2.

	Run 1 - Pronounced Speech							
Participant	Up	Down	Right	Left				
sub-01	10	10	10	10				
sub-02	10	10	10	10				
sub-03	10	10	10	10				
sub-04	10	10	10	10				
sub-05	10	10	10	10				
sub-06	10	10	10	10				
sub-07	10	10	10	10				
sub-08	10	10	10	10				
sub-09	10	10	10	10				
sub-10	10	10	10	10				
	Run 2 - Inner Speech				Run 3 - Inner Speech			
Participant	Up	Down	Right	Left	Up	Down	Right	Left
sub-01	10	10	10	10	10	10	10	10
sub-02	10	10	10	10	10	10	10	10
sub-03	10	10	10	10	10	10	10	10
sub-04	10	10	10	10	10	10	10	10
sub-05	10	10	10	10	10	10	10	10
sub-06	10	10	10	10	10	10	10	10
sub-07	10	10	10	10	10	10	10	10
sub-08	10	10	10	10	10	10	10	10
sub-09	10	10	10	10	10	10	10	10
sub-10	10	10	10	10	10	10	10	10
	Run 4 - Visualized Condition				Run 5 - Visualized Condition			
Participant	Up	Down	Right	Left	Up	Down	Right	Left
sub-01	10	10	10	10	10	10	10	10
sub-02	10	10	10	10	10	10	10	10
sub-03	10	10	10	10	10	10	10	10
sub-04	10	10	10	10	10	10	10	10
sub-05	10	10	10	10	10	10	10	10
sub-06	10	10	10	10	10	10	10	10
sub-07	10	10	10	10	10	10	10	10
sub-08	10	10	10	10	10	10	10	10
sub-09	10	10	10	10	10	10	10	10
sub-10	10	10	10	10	10	10	10	10

Supplementary Table 3. Distribution of trials in session 3. * Check the "Ad-Hoc Correction" Section for further details of the uneven number of trials in Participant sub-03.

	Run 1 - Pronounced Speech							
Participant	Up	Down	Right	Left				
sub-01	5	5	5	5				
sub-02	10	10	10	10				
sub-03	5	5	5	5				
sub-04	10	10	10	10				
sub-05	10	10	10	10				
sub-06	7	7	7	7				
sub-07	10	10	10	10				
sub-08	5	5	5	5				
sub-09	10	10	10	10				
sub-10	10	10	10	10				
	Run 2 - Inner Speech				Run 3 - Inner Speech			
Participant	Up	Down	Right	Left	Up	Down	Right	Left
sub-01	5	5	5	5	5	5	5	5
sub-02	10	10	10	10	10	10	10	10
sub-03	5	5	5	5	10*	10*	10*	10*
sub-04	10	10	10	10	10	10	10	10
sub-05	10	10	10	10	10	10	10	10
sub-06	7	7	7	7	7	7	7	7
sub-07	10	10	10	10	10	10	10	10
sub-08	5	5	5	5	5	5	5	5
sub-09	10	10	10	10	10	10	10	10
sub-10	10	10	10	10	10	10	10	10
	Run 4 - Visualized Condition				Run 5 - Visualized Condition			
Participant	Up	Down	Right	Left	Up	Down	Right	Left
sub-01	5	5	5	5	5	5	5	5
sub-02	10	10	10	10	10	10	10	10
sub-03	0*	0*	0*	0*	5	5	5	5
sub-04	10	10	10	10	10	10	10	10
sub-05	10	10	10	10	10	10	10	10
sub-06	7	7	7	7	7	7	7	7
sub-07	10	10	10	10	10	10	10	10
sub-08	5	5	5	5	5	5	5	5
sub-09	10	10	10	10	10	10	10	10
sub-10	10	10	10	10	10	10	10	10
